# Immunomodulation by Different Types of N-Oxides in the Hemocytes of the Marine Bivalve *Mytilus galloprovincialis*

**DOI:** 10.1371/journal.pone.0036937

**Published:** 2012-05-11

**Authors:** Caterina Ciacci, Barbara Canonico, Dagmar Bilaniĉovă, Rita Fabbri, Katia Cortese, Gabriella Gallo, Antonio Marcomini, Giulio Pojana, Laura Canesi

**Affiliations:** 1 Dipartimento di Scienze della Terra, della Vita e dell’Ambiente - DISUAN, Università degli Studi di Urbino “Carlo Bo”, Urbino, Italy; 2 Dipartimento di Scienze Ambientali, Informatica e Statistica, Università Ca’ Foscari di Venezia, Venezia, Italy; 3 Dipartimento di Scienze della Terra, dell’Ambiente e della Vita, DISTAV, Università di Genova, Genova, Italy; 4 Dipartimento di Medicina Sperimentale - DIMES, Università di Genova, Genova, Italy; INRA, France

## Abstract

The potential toxicity of engineered nanoparticles (NPs) for humans and the environment represents an emerging issue. Since the aquatic environment represents the ultimate sink for NP deposition, the development of suitable assays is needed to evaluate the potential impact of NPs on aquatic biota. The immune system is a sensitive target for NPs, and conservation of innate immunity represents an useful basis for studying common biological responses to NPs. Suspension-feeding invertebrates, such as bivalves, are particularly at risk to NP exposure, since they have extremely developed systems for uptake of nano and microscale particles integral to intracellular digestion and cellular immunity. Evaluation of the effects of NPs on functional parameters of bivalve immunocytes, the hemocytes, may help understanding the major toxic mechanisms and modes of actions that could be relevant for different NP types in aquatic organisms.In this work, a battery of assays was applied to the hemocytes of the marine bivalve *Mytilus galloprovincialis* to compare the *in vitro* effects of different n-oxides (n-TiO_2_, n-SiO_2_, n-ZnO, n-CeO_2_) chosen on the basis of their commercial and environmental relevance. Physico-chemical characterization of both primary particles and NP suspensions in artificial sea water-ASW was performed. Hemocyte lysosomal and mitochondrial parameters, oxyradical and nitric oxide production, phagocytic activity, as well as NP uptake, were evaluated. The results show that different n-oxides rapidly elicited differential responses hemocytes in relation to their chemical properties, concentration, behavior in sea water, and interactions with subcellular compartments. These represent the most extensive data so far available on the effects of NPs in the cells of aquatic organisms. The results indicate that *Mytilus* hemocytes can be utilized as a suitable model for screening the potential effects of NPs in the cells of aquatic invertebrates, and may provide a basis for future experimental work for designing environmentally safer nanomaterials.

## Introduction

The potential toxicity of engineered nanoparticles (NPs) for humans and the environment represents an emerging issue, due to the continuous development and production of manufactured nanomaterials [1], [2]. Since NPs tend to end up in waterways, their uptake and effects in the aquatic biota represent a major concern [3]–[5]. Apart from traditional ecotoxicity testing, it has been underlined that more specific assays like immunotoxicity, genotoxicity, oxidative stress, may help understanding the major toxic mechanisms and modes of actions that could be relevant for different NP types also in aquatic organisms [6]. According to [7], invertebrate tests are well suited to generate reproducible and reliable nanotoxicity data: invertebrates represent about 95% of animal species, have an important ecological role, and represent potential transfer of NPs through food chains. In these organisms, potential routes of exposure are ingestion or entry through epithelial surfaces; moreover, they have highly developed processes for cellular internalization of nano- and micro-scale particles (endocytosis and phagocytosis), that are integral to key physiological functions such as intracellular digestion and cellular immunity [8].

The immune system is considered as sensitive target for the effect of NPs in mammals [9], [10] and potential interactions of NPs with immune cells represent a major issue for both therapeutic use and possible detrimental effects on human health. Since different types of NPs may induce immunostimulation or immunosuppression in different experimental models, immunotoxicity tests have been widely applied in an attempt to design representative and robust assays that can be utilized for effective screening of NP-induced immunomodulatory effects [10]–[13].

Invertebrates lack adaptive immunity: however, they are endowed with a potent innate immune system [14]. Conservation of the general mechanisms of innate immunity from invertebrates to mammals is a key feature that represents an useful basis for studying common biological responses to environmental contaminants, including NPs.

Bivalve mollusks are a relevant ecological group, widespread in freshwater, estuarine and marine environments, with many edible species, and widely utilized to evaluate the effects of different contaminants. Increasing evidence support the hypothesis that bivalves may represent a significant target group for NP toxicity [15]. In these organisms, the blood cells, the hemocytes, are responsible for cell-mediated immunity through phagocytosis and various cytotoxic reactions [16]. Although bivalve hemocytes are extremely heterogeneous, in the marine mussel *Mytilus galloprovincialis* granular hemocytes represent the dominant cell type and are characterised by high phagocytic activity and capacity for oxyradical production [17]. Responses of mussel hemocytes to bacterial signals, cytokines, hormones, as well as to a variety of contaminants, have been largely characterized ([15] and references quoted therein). In these cells, the immune function is modulated by conserved components of kinase-mediated cell signaling [18].

We have previously shown that *in vitro* exposure to NPs (both carbon based and n-oxides), in the same concentration range as that generally utilized in mammalian cells, induced significant changes in immune parameters in mussel hemocytes through modulation of stress activated p38 MAPK [19], [20]. The results suggested that distinct responses (resulting in immunotoxicity/immunomodulation) may be elicited by different types of NPs. In this work, in order to investigate the possible specificity of the hemocyte response NPs, a battery of functional assays was applied to compare the effects of different n-oxides (n-TiO_2_, n-SiO_2_, n-ZnO, n-CeO_2_) with a narrow size distribution (declared particle size in the 15–42 nM range), chosen on the basis of their commercial and environmental relevance. Physico-chemical characterization of both primary particles and NP suspensions in artificial sea water (ASW) was performed and lysosomal and mitochondrial parameters, oxyradical production and phagocytic activity, as well as NP uptake, were evaluated.

## Results

### Characterization of Primary NPs and NP Suspensions in ASW

Characterization of the primary particles was performed in order to verify the declared properties. and the results are summarized in Table 1. The actual average sizes resulted to be quite different from the declared ones, as inferred from Trasmission Electron Microscopy-TEM analysis (Table 1 and Fig. S1). In particular, a wide size distribution was found for n-ZnO. A wide range of surface areas was also found, from 14 m^2^/g for n-ZnO to 226 m^2^/g for n-SiO_2_. All n-oxides showed a well defined crystal structure, apart from nanosilica, which resulted substantially amorphous. Experimentally determined pore volumes were very similar for all n-oxides (0.1 ml/g), apart from n-SiO_2_ (0.7 ml/g). The declared high purity was confirmed for all selected nanopowders.

**Table 1:** Primary physical and chemical properties of selected NPs.

NP type	Crystal structure 1	Shape 1	Size distribution(nm) 1	Surface area(m^2^/g) 2	Pore volume(mL/g) 2	Surface chemistry 3	Chemical composition 1	Purity (%) 4
TiO_2_	Anatase/Rutile	Irregular	15–60	61	0.1	uncoated	Ti, O	>99.5
ZnO	Cubic/tetragonal/orthorhombic	Polyhedral	10–2000	14	0.1	uncoated	Zn, O	>99
SiO_2_	Amorphous	Irregular	5–30	226	0.7	uncoated	Si, O	>99
CeO_2_	Fluorite	Irregular	5–20	45	0.1	uncoated	Ce, O	>99

1 –by TEM, TEM-EDX, SAED.

2 –by BET.

3 –declared by the supplier.

4 –by ICP-OES.

The behaviour of different n-oxides in suspension in the ASW utilized for exposure experiments was investigated by Dynamic Light Scattering-DLS analysis. Table 2 summarizes the size distribution of selected NP suspensions in ASW. The actual size distribution was determined in NP suspensions in ASW at concentrations of 50 and 200 µg/ml (1 mg/l for silica, due to its intrinsic low scattering signal) after 15 min, 1 h and 24 h from sonication. All n-oxides showed a strong tendency to agglomeration, a natural process with NPs not stabilized by interparticle repulsive forces, giving size distributions from 18 (n-SiO_2_) to 126 (n-TiO_2_) times their average primary particle size, even at the lowest examined concentration. Although such agglomerates showed increases in size within the first hour from sonication, they should be considered quite stable, taking into account that an high energy dispersion procedure (probe sonication at 100 W) was applied in order to disperse them in the testing medium.

**Table 2:** Average size distributions of selected NP suspensions in ASW at different times after sonication, as determined by Dynamic Light Scattering (DLS).

NP type	Concentration in ASW	Average size distribution (± Standard Deviation) with time (nm)		
		After 15 min	After 1 h	After 24 h
TiO2	200 µg/ml	4195 (±1433)	5514 (±4199)	5846 (±4568)
	50 µg/ml	2659 (±1957)	3982 (±3361)	4440 (±3765)
ZnO	200 µg/ml	2852 (±1550)	3765 (±2337)	4056 (±2193)
	50 µg/ml	2193 (±1525)	3036 (±2332)	3467 (±2547)
CeO2	200 µg/ml	2665 (±1425)	4872 (±4039)	5034 (±4479)
	50 µg/ml	1906 (±1140)	2919 (±2086)	3438 (±3118)
SiO2	1 mg/ml	178 (±105)	180 (±98)	208 (±103)

### Effects on Hemocyte Lysosomal Membrane Stability, Lysozyme Release and Phagocytosis

The effects of different n-oxides on *Mytilus* immune cells were evaluated using a battery of assays, utilizing different exposure times (from 30 min to 4 h) and conditions optimized for each assay to avoid NP interference. No significant changes in hemocyte viability were observed in the different experimental conditions (data not shown).

**Figure 1:**
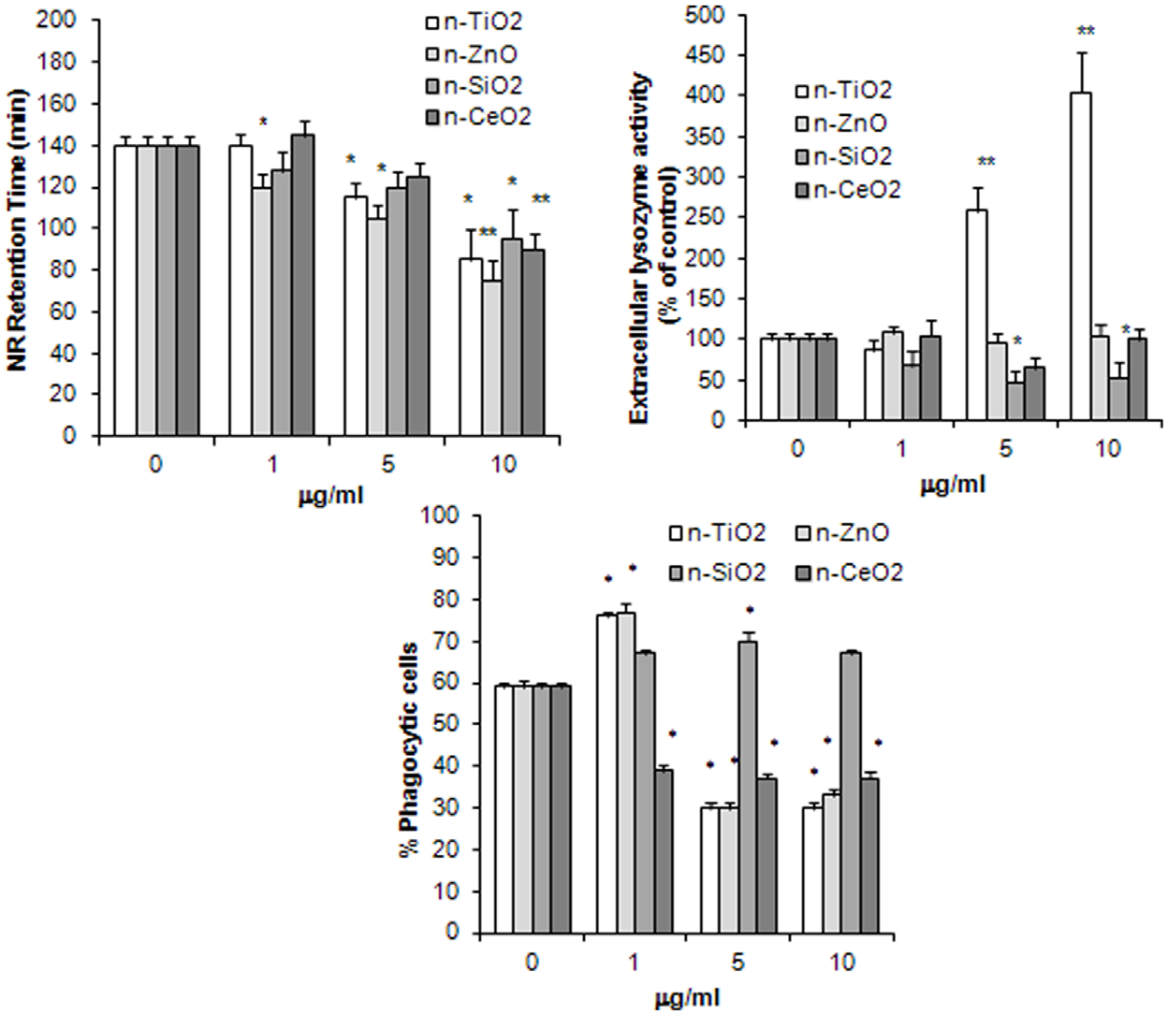
Effects of n-TiO_2_, n-SiO_2_, n-ZnO, n-CeO_2_ on functional parameters of mussel hemocytes. A) lysosomal membrane stability (LMS); B) extracellular lysozyme release; C) phagocytic activity. Hemocytes were exposed to different concentrations of n-oxides (1, 5, 10 µg/ml) and different parameters were evaluated as described in Methods. Data, representing the mean±SD of four experiments in triplicate, were analysed by ANOVA followed by Tukey’s post hoc test. A, B: *  =  P≤0.05; **  =  P≤0.01, all treatments *vs* controls; C: *  =  P≤0.01, all treatments *vs* controls.

Lysosomal membrane stability (LMS), lysozyme release and phagocytic activity were evaluated in hemocytes incubated with different concentrations of n-TiO_2_, n-SiO_2_, n-ZnO, n-CeO_2_ (1, 5 and 10 µg/ml) and the results are reported in Fig. 1. Hemocyte incubation for 30 min with all n-oxides affected hemocyte LMS. As shown in Fig. 1A, a clear dose-dependent decrease was induced by n-ZnO at all the concentrations tested (–46% with respect to control values at 10 µg/ml; p≤0.01). N-TiO_2_ induced lysosomal destabilization at 5 and 10 µg/ml (–18 and −39%, respectively; p≤0.05), whereas n-SiO_2_ and n-CeO_2_ were effective only at the highest concentration tested (−32 and −36%, respectively; p≤0.05 and p≤0.01).

N-TiO_2_ stimulated lysozyme release at both 5 and 10 µg/ml (up to four-fold increase with respect to controls at the highest concentration tested; p≤0.01), whereas both n-ZnO and n-CeO_2_ were ineffective (Fig. 1B). On the other hand, n-SiO_2_ induced a decrease in extracellular lysozyme activity at higher concentrations (about −50% with respect to controls; p≤0.05).

Both n-TiO_2_ and n-ZnO significantly increased phagocytosis of Neutral Red-conjugated zymosan particles at the lowest concentration tested (about +30% with respect to controls; p≤0.01), whereas higher concentrations induced a dramatic decrease in phagocytic activity (−50%; p≤0.01) (Fig. 1C). N-SiO_2_ did not affect phagocytosis, except for a small increase (+18%; p≤0.01) at 5 µg/ml. On the other hand, n-CeO_2_ inhibited phagocytosis at all the concentrations tested (about −35% with respect to controls; p≤0.01).

### Effects on Oxyradical and Nitric Oxide Production

**Figure 2:**
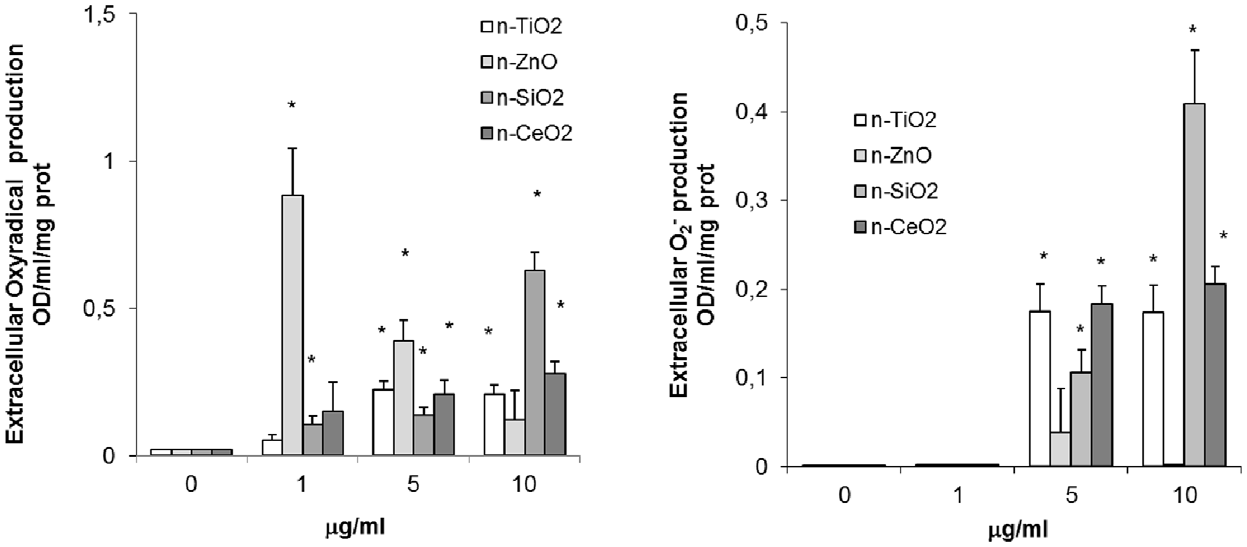
Effects of n-TiO_2_, n-SiO_2_, n-ZnO, n-CeO_2_ on extracellular oxyradical production by mussel hemocytes. A) Total oxyradical production; B) Superoxide anion (O_2_^−^) production. Hemocytes were exposed for 30 min to different concentrations of n-oxides (1, 5, 10 µg/ml) and total extracellular oxyradical production was evaluated as cytochrome c reduction as described in Methods (A). In a parallel set of samples, superoxide dismutase-SOD (300 Units/ml) was included to allow specific evaluation of superoxide (O_2_^−^) generation (B). Data, representing the mean±SD of four experiments in triplicate, were analysed by ANOVA followed by Tukey’s post hoc test. *  =  P≤0.01, all treatments *vs* controls.

Different n-oxides stimulated total extracellular oxyradical (or reactive oxygen species-ROS) production, evaluated as cyt *c* reduction (Fig. 2A). The effect of n-ZnO was significant at lower concentrations, and maximal at 1 µg/ml (p≤0.01). N-SiO_2_ induced a dose-dependent rise in ROS production at all the concentrations tested (p≤0.01). Smaller effects were observed with both n-TiO_2_ and n-CeO_2_, that were significant at 5 and 10 µg/ml (p≤0.01) When extracellular superoxide (O_2_^-^) was evaluated as SOD-inhibitable ROS production, n-SiO_2_, n-TiO_2_ and n-CeO_2_ induced significant increases at both 5 and 10 µg/ml (p≤0.01), with n-SiO_2_ showing the strongest effect at the highest concentration, whereas n-ZnO was ineffective (Fig. 2B).

**Figure 3:**
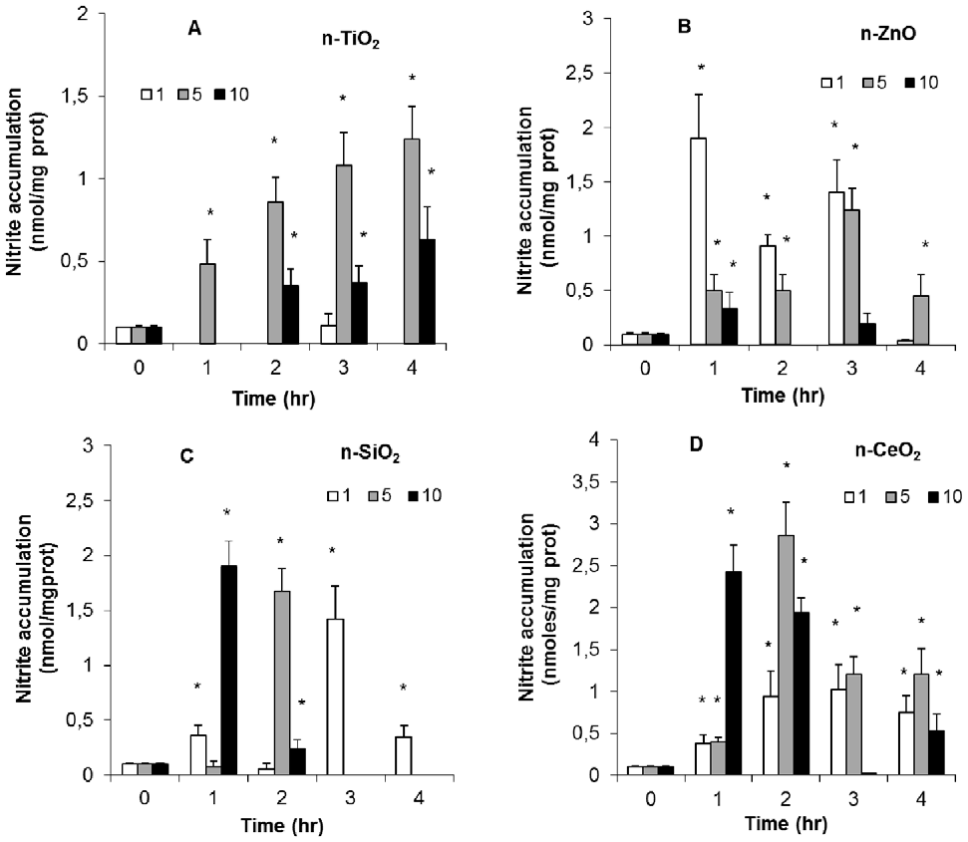
Effects of n-TiO2, n-SiO2, n-ZnO, n-CeO2 on NO production by mussel hemocytes. Hemocytes were exposed to different concentrations of n-oxides (1, 5, 10 g/ml) for different times (from 1 to 4 hr) and NO production was evaluated as nitrite accumulation by the Griess reaction as described in Methods. A) n-TiO2; B) n-SiO2; C) n-ZnO, D) n-CeO2. Data, representing the mean�SD of four experiments in triplicate, were analysed by ANOVA followed by Tukey's post hoc test. * = P=0.05, all treatments vs controls..

Nitric oxide-NO production was evaluated as nitrite accumulation in hemocytes incubated with n-oxides for different periods of time (from 1 to 4 h) (Fig. 3). Average NO production by control hemocytes was about 0.12±0.02 nmoles nitrite/mg protein throughout the experimental period. With n-TiO_2_, the lowest concentration was ineffective, whereas at higher concentrations a time-dependent increase in NO production was observed, with 5 µg/ml producing the strongest effects (p≤0.05) (Fig. 3A). For n-ZnO, an inverse relationship between particle concentration and nitrite accumulation was observed (Fig. 3B). In particular, 1 µg/ml n-ZnO induced the largest rise in NO production between 1 and 3 h incubation (p≤0.05). For both n-SiO_2_ and n-CeO_2_, maximal nitrite accumulation was observed with the highest concentration at shorter times of incubation, whereas lower concentrations were effective at longer incubation times (p≤0.05) (Fig. 3C and 3D).

### Effects on Mitochondria

**Figure 4:**
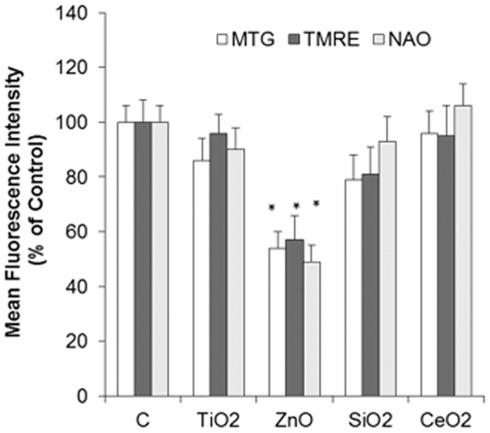
Effect of n-TiO_2_, n-SiO_2_, n-ZnO, n-CeO_2_ on mitochondria of mussel hemocytes. Hemocytes were exposed to different n-oxides (10 µg/ml) for 45 min and subsequently loaded with the fluorescent dyes Mito Tracker MT (for mitochondrial mass/number), with TMRE (for membrane potential Δψm) and with NAO for cardiolipin content and fluorescence intensities were evaluated by flow cytometry as described in methods. Data are reported as Mean Fluorescence Intensities (percent) with respect to controls. Data, representing the mean±SD of three experiments, were analysed by ANOVA followed by Tukey’s post hoc test. *  =  P≤0.05, all treatments *vs* controls.

The effects of hemocyte incubation with different n-oxides (45 min, 10 µg/ml) on mitochondrial parameters were evaluated by Flow Cytometry utilizing specific fluorescent dyes for active mitochondria (MitoTracker Green), mitochondrial membrane potential Δψ_m_ (Tetramethylrhodamine, ethyl-ester perchlorate -TMRE) and Cardiolipin oxidation in the inner mitochondrial membrane (10-nonyl-acridine orange-NAO), and the results are reported in Fig. 4. The results indicate that n-ZnO induced a significant decrease in mitochondrial mass/number (−36% with respect to controls, p≤0.05) and membrane potential ΔΨ (−40%, p≤0.05), as well as an increase in cardiolipin oxidation (−51%, p≤0.05). On the other hand, the other types of n-oxides did not affect mitochondrial parameters.

### Transmission Electron Microscopy (TEM)

**Figure 5:**
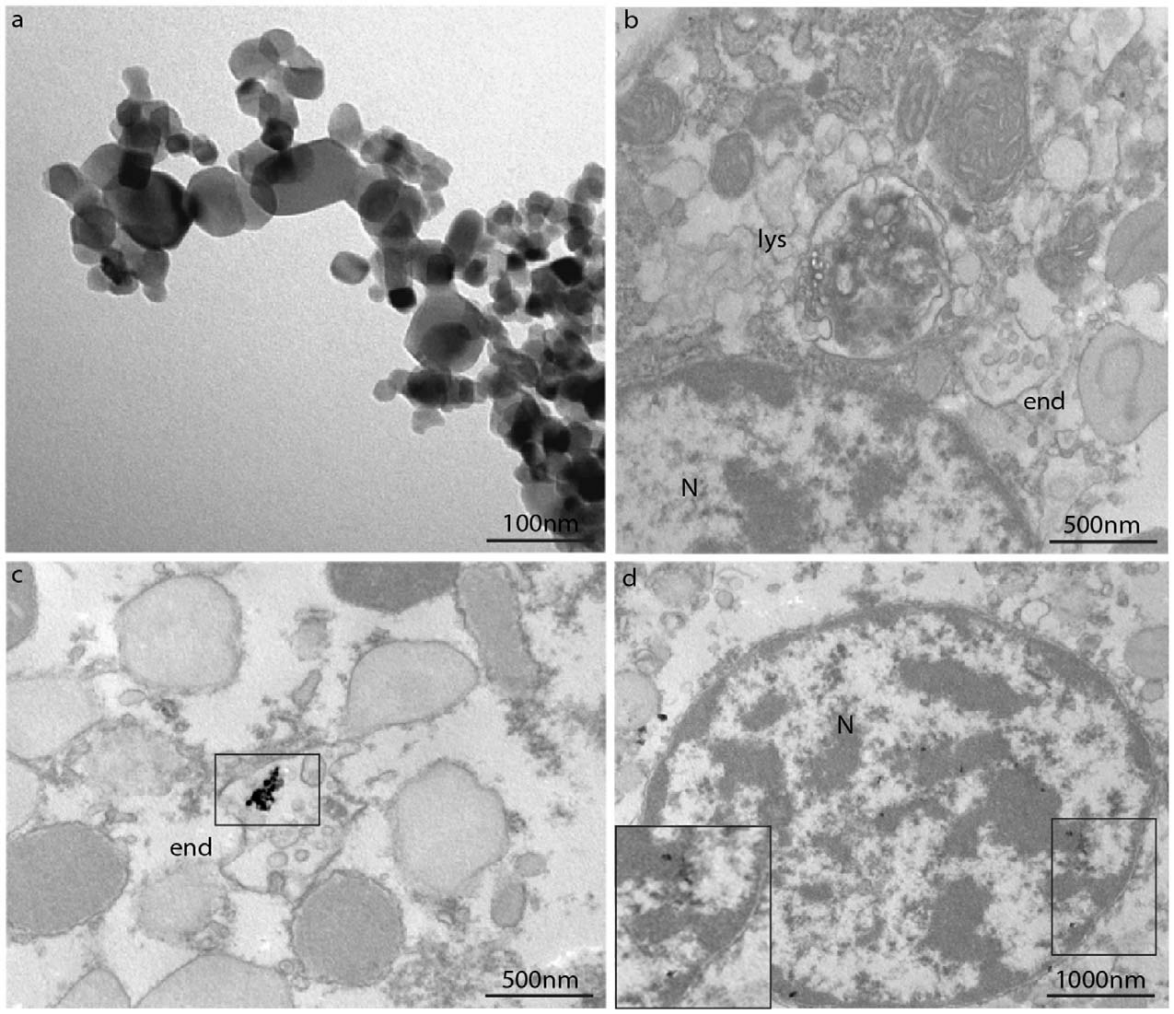
TEM images of n-TiO_2_ suspensions and their intracellular localisation in mussel hemocytes. Hemocytes were incubated with n-TiO_2_ (10 µg/ml in ASW) for 30 and 60 min as described in Methods. A) n-TiO_2_ suspensions (10 µg/ml in ASW); B) Control cells, showing lysosomal (lys) and endosomal structures (end); C) n-TiO_2_ (30 min), showing the presence of TiO_2_ agglomerates within an endosome (end). D) n-TiO_2_ (60 min). The square indicates the presence of n-sized TiO_2_ within the nucleus (N) (see enlargement at the bottom left).

**Figure 6:**
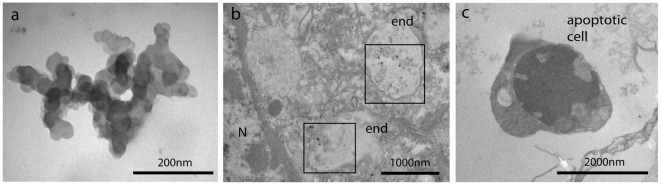
TEM images of n-ZnO suspensions (10 µg/ml in ASW) (A) and their intracellular localisation in mussel hemocytes (B–C). Hemocytes were incubated with n-ZnO (10 µg/ml in ASW) for different periods of time as described in Methods. B) n-ZnO (30 min). Squares indicate the presence of n-sized ZnO particles within multivesicular endosomes (end). C) n-ZnO (60 min). An apoptotic cell is shown.

Observations on n-TiO_2_ and n-ZnO suspensions in ASW at the highest concentration utilized in exposure experiments (10 µg/ml) and their possible uptake by mussel hemocytes at different times of exposure (30 and 60 min) were carried out by TEM, and representative images are reported in Figs. 5 and 6. As shown in Fig. 5A, in the n-TiO_2_ suspension agglomerates of different sizes (500 nm-1 µm) were observed. Control hemocytes are shown in Fig. 5B and hemocytes incubated with n-TiO_2_ in Figs. 5C and 5D. Exposure to n-TiO_2_ did not apparently affect the hemocyte morphology. n-TiO_2_ agglomerates of 200–250 nm size were observed within endosomes at 30 min incubation (Fig. 5C). At 60 min, nanosized particles were observed within the nucleus (Fig. 5D).

In Fig. 6 are reported TEM images of n-ZnO suspensions, indicating the presence of agglomerates of hundreds nm (Fig. 6A). In hemocytes exposed to n-ZnO, nanosized ZnO particles were found within endosomes at 30 min incubation (Fig. 6B). Moreover, at 60 min of exposure, apoptotic cells could be observed (Fig. 6C).

## Discussion

### Characterization of Different n-oxides

Primary particles revealed a wide range of average size distributions and surface areas, comparable pore volumes, except for n-SiO_2_, and similar purity for different types of n-oxides (Table 1). Moreover, behavior of n-oxide suspensions in ASW at different concentrations and times after sonication was investigated by DLS analysis, indicating a general strong tendency to formation of stable agglomerates of micrometric size for all n-oxides, except for n-SiO_2_ (Table 2).

Although the size obtained by DLS is usually greater than that measured by other techniques, like TEM, BET, etc., during DLS measurements there is a tendency of particles to aggregate in the aqueous state, so this method gives the sizes of clustered particles rather than individual particles. Moreover, NP concentrations higher than those utilized in exposure experiments were utilized for DLS analysis, due to intrinsic low scattering signal. However, the DLS system also affords the option of considering the average hydrodynamic diameter of the particles in terms of number and under conditions that more closely resemble the exposure conditions, so it can provide an idea of the particle suspension stability with respect to time and medium [21]. When suspensions of n-TiO_2_ or n-ZnO at concentrations utilized in cell exposure experiments (10 µg/ml in ASW) were analysed by TEM, agglomerates of smaller size (hundreds nm), were observed (Fig. 5 and 6). The observed formation of NP agglomerates in ASW cannot of course be predictive of the behaviour of NPs in natural waters, in particular in coastal and estuarine waters that represent the natural environment of mussels, and that are subjected to fluctuations in salinity and concentrations of organic substances.

### Effects of Different n-oxides on Hemocyte Parameters

In our experimental conditions, suspensions of all the n-oxides tested significantly affected functional parameters of *Mytilus* hemocytes, with distinct effects or to a different extent for different endpoints. The observed responses were comparable with those induced by bacterial signals, hormones, or other contaminants [15], [18].

All n-oxides induced moderate but significant decreases in lysosomal membrane stability (LMS), a sensitive parameter of cellular stress in bivalves, with ZnO>TiO_2_ = SiO_2_>CeO_2_. The effect was apparently unrelated to the primary particle size or to the size of agglomerates of NP suspensions in ASW. Only n-TiO_2_ also induced a significant increase in lysozyme release at higher concentrations. Stimulation of total extracellular ROS production was also observed, with ZnO>SiO_2_>TiO_2_ = CeO_2_. With n-ZnO an inverse relationship between concentration and oxyradical production was observed, whereas other n-oxides induced a dose-dependent effect. Moreover, when the SOD-inhibitable ROS production was determined, the effects of all n-oxides, except for n-ZnO, were mainly due to production of O_2_^−^, with SiO_2_>TiO_2_ = CeO_2_. All n-oxides also induced nitrite accumulation, indicating stimulation of NO production, with distinct concentration- and time-dependent effects depending on the NP type. Both n-TiO_2_ and n-ZnO showed a biphasic effect on phagocytosis of NR-conjugated zymosan particles, with stimulation at the lowest concentration and inhibition at higher concentrations. A small increase in phagocytic activity was also observed with n-SiO_2_, that was however ineffective at higher concentrations. On the other hand, n-CeO_2_ inhibited phagocytosis at all the concentrations tested. Since phagocytosis is commonly used as a proxy for immunocompetence in bivalves [22], data on hemocyte phagocytic activity may reflect the overall impact of NPs on the immune function. However, the possibility that, at higher concentrations, n-TiO_2_ and n-ZnO agglomerates may specifically compete with zymosan particles for phagocytosis by the hemocytes must be considered.

With regards to the effects of each n-oxide type on hemocyte functional parameters, n-SiO_2_ scarcely affected LMS and phagocytosis in comparison with other n-oxides, whereas it was a powerful inducer of ROS (O_2_^−^ in particular) and NO production, indicating inflammatory processes. Such an effect may be due to stronger interactions with cellular membranes of NPs with larger surface area and pore volume, as well as to formation of smaller agglomerates in ASW (see Tables 1 and 2).

The present work reports the first data on the cellular effects of n-CeO_2_ in aquatic organisms. N-CeO_2_ has been developed as a fuel additive, and it is likely to be released into waste waters and the atmosphere and thus be distributed widely in the aquatic environment [23]. Ecotoxicity of n-CeO_2_ in aquatic organisms is generally low (EC_50_ in the mg/l range from bacteria to fish) ([24] and reference quoted therein). In mammalian cells, the toxicity of n-CeO_2_ is still controversial, with often conflicting results depending on the experimental model, especially regarding the oxidant/antioxidant effect [23]. Interactions with plasma membranes may involve an oxidative response due to reduction of Ce(IV) to Ce(II). On the other hand, n-CeO_2_ is considered a unique nanomaterial because it exhibits anti-inflammatory properties, potentially acting as a ROS scavenger with superoxide dismutase-like activity [25]. However, such as an effect may occur in the cytosol and mitochondria, whereas toxicity seems to be associated with cellular uptake and localization in the acidic lysosomal matrix, especially in phagocytic cells [25], [26]. This is highly important as some nanomaterials may display different behavior and exert either a beneficial (antioxidant) or toxic (oxidant) effect, depending not only on particle charge in different experimental media [24] but also on the pH of the subcellular compartment where they localize [25]. Our results show in mussel hemocytes that n-CeO_2_ induced ROS and NO production, at the same time inhibiting the phagocytic activity, this indicating that n-CeO_2_ may have inflammatory and/or immunosuppressive effects. The effects of nanoceria with mussel cells, in terms of subcellular localisation and pro-oxidant/antioxidant effects, require further investigation.

N-ZnO showed the strongest effects on LMS. In bivalve hemocytes, reduction of lysosomal stability is linked with impaired cellular immunity [8], [27]. At the lowest concentration tested, when a small decrease in LMS was observed, ROS and NO production, as well as phagocytic activity, were increased, indicating immunostimulation. On the other hand, at increasing concentrations, stronger lysosomal destabilization was associated with inhibition of phagocytosis and smaller, if any, ROS/NO production. Moreover, at the highest concentration, FC analysis revealed specific effects on mitochondria, such as decreases in mitochondrial mass/number and membrane potential, as previously observed in hemocytes treated with carbon black NPs [19]. N-ZnO also induced mitochondrial cardiolipin oxidation; moreover, increases in Annexin V binding at the plasma membrane was observed (data not shown). Overall, the results indicate that n-ZnO can induce pre-apoptotic processes in mussel hemocytes, as confirmed by the presence of apoptotic cells visualized by TEM. The toxicity of n-ZnO on marine organisms was shown to be influenced significantly by the release of Zn^2+^ ions [28], [29]. Actually, the effects of higher concentrations of n-ZnO on immune parameters of *Mytilus* hemocytes are comparable with those elicited by ZnCl_2_ [30], [31]. Moreover, the effects of n-ZnO (lysosomal destabilization, mitochondrial injury) were similar to those observed in mammalian cells, including macrophages [32], [33]. This toxicity was directly related not only to particle dissolution and release of toxic Zn^2+^ in the cell culture medium, but also to particle uptake and Zn^2+^ dissolution within the acidic endosomal/lysosomal compartments. In order to get a first insight into the possible uptake and intracellular localization of n-oxides in mussel hemocytes, TEM observations were performed in cells exposed to n-ZnO. Endosomal localization of ZnO nanosized particles was observed in mussel hemocytes, supporting this hypothesis.

TiO_2_ is the most widely produced nanomaterial [34], and it may reach environmental concentrations to pose a significant threat to aquatic ecosystems. *In vivo* exposure to n-TiO_2_ in the low mg/l range showed adverse effects on different aquatic organisms [35], [36], [37]. Our *in vitro* data show that, in the same concentration range, n-TiO_2_ significantly affected all the parameters measured in mussel hemocytes. At the lowest concentration tested, n-TiO_2_ stimulated the phagocytic activity without significantly affecting other endpoints; at increasing concentrations, increased lysosomal destabilization, lysozyme release, oxidative burst and NO production were associated with a decrease in phagocytosis. TEM analysis indicated the presence of TiO_2_ agglomerates in endocytic vacuoles at 30 min (but also at 15 min, data not shown); at longer times of incubation nanosized material was observed within the nuclear compartment.

With regards to the *in vitro* effects of n-TiO_2_ and n-SiO_2_ on mussel hemocytes, the result largely confirm and extend previous observations in the same experimental conditions [20]. With respect to the previous work, discrepancies were in fact observed only for the effects of n-TiO_2_ on LMS and of n-SiO_2_ on lysozyme release. Such differences may be partly due to biological factors; this could explain the stronger effects of n-TiO_2_ on LMS observed in the present work. On the other hand, distinct effects on lysozyme release were observed for n-SiO_2_. However, in the present work, a nanosilica with similar size and physico-chemical declared properties, but obtained from a different source, was utilized. Differences in the properties of the two types of nanosilica could also partially explain their distinct effects on different biological endpoints. Overall, the results strongly support the view that in invertebrate hemocytes, the evaluation of a single functional parameter cannot be considered as fully representative of immunocompetence. Our data strongly support the view that the application of a battery of functional assays is needed to evaluate the overall impact of environmental stressors, including NP exposure, on bivalve immune function [22].

Taken together, the results indicate that the immunomodulatory effects of different n-oxides on mussel hemocytes mainly depend not only on the concentration, but also on particle chemistry and behaviour in ASW. Although the results of *in vitro* experiments are not necessarily predictive of *in vivo* effects, exposure to different types on NPs in both freshwater and marine bivalves has been shown to both affect hemocyte parameters and to induce stress responses in different tissues, indicating adverse effects at the organism level [35], [38]. Moreover, exposure to n-TiO_2_ in the low µg/l range confirm the effects of n-TiO_2_ on *Mytilus* immune parameters *in vivo* (Canesi et al., manuscript in preparation).

Overall, the results obtained in *Mytilus* hemocytes represent the most extensive data so far available on the effects of NPs in the cells of aquatic organisms, and indicate that hemocytes can represent a sensitive *in vitro* model for the rapid screening of the cellular effects of different NPs. Moreover, the results give an insight into the possible mechanisms of action of different NP types, their interactions with different cellular compartments and effects on the innate immune response. These data add information on the potential impact of commercial NPs in aquatic organisms and can provide a basis for future experimental work related to designing safer nanomaterials.

## Materials and Methods

### NP Characterization

Nanosized Titanium Dioxide P25 (declared size: 21 nm) was provided from Degussa Evonik (Essen, Germany), with a declared purity of >99.5%. Nanosized silica (declared size: 20 nm) and nanosized zinc oxide (declared size: 42 nm) were kindly provided from Kristoph Klein (European Commission - Joint Research Centre, Ispra, I). Nanosized Cerium oxide (declared size: 15–30 nm) was kindly provided by Prof. Enrico Bergamaschi (University of Parma, I).

These n-oxides were characterized by a combination of analytical techniques. Mean average size, shape and crystal structure of primary particles were determined by Transmission Electron Microscope (TEM) analysis on a Jeol (Tokyo, Japan) 3010 transmission electron microscope operating at 300 kV. TEM images of different n-oxides are shown in Fig. S1. Surface area and pore volume were obtained by nitrogen adsorption on a Micrometrics ASAP2000 Accelerated Surface Area and Porosimetry System at an adsorption temperature of −196°C, after pretreating the sample under high vacuum at 300°C for 2 h [39]. Table 1 summarizes the results obtained for primary particle characterization.

Stock suspensions of investigated NPs were freshly prepared for DLS analysis in artificial sea water (ASW) (36‰ salinity) prepared according to the ASTM E 724–98 protocol [40], and filtered through a 0.45 µm Teflon® filter. NP suspensions were prepared at a concentration of 0.05–1 mg/ml, then sonicated for 15 min at 100 W and 50% on/off cycle with a UP200S Hielscher Ultrasonic Technology (Teltow, Germany) in an ice/water bath. Dynamic Light Scattering (DLS) analysis was performed with a Submicron Particle Sizer Nicomp 370 DLS (Santa Barbara, CA, USA), equipped with a 5 mW He-Ne laser, 632.8 nm laser diode and a photodiode detector set at 90°. Table 2 summarizes the size distribution of selected NP suspensions in ASW.

### Animals, hemolymph Collection and Hemocyte Treatment

Mussels (*Mytilus galloprovincialis* Lam.) 4–5 cm long, obtained from a mussel farm at Arborea (OR, Italy) were kept for 1–3 days in static tanks containing 36 ‰ salinity ASW, 1 l/mussel, at 16°C. Sea water was changed daily. Hemolymph was extracted from the posterior adductor muscle sinus, using a sterile 1 ml syringe with a 18 G1/2″ needle. Hemolymph collection and hemocyte treatments were carried out as previously described [19]. For each sample, hemolymph from 8–10 individuals was filtered through a sterile gauze and pooled in 50 ml Falcon tubes at 4°C. Stock suspensions of NPs in ASW (10 µg/ml) were prepared by sonication as for DLS analysis and immediately added to the samples in order to reach the desired concentrations. Hemocyte suspensions or hemocyte monolayers, depending on the endpoint measured, were incubated at 16°C with different concentrations of NP suspensions (1, 5, 10 µg/ml) for different periods of time (from 30 min to 4 hrs), as indicated in each experiment. Different times of incubation with NP suspensions were utilised for measuring each endpoint in order to optimise the *in vitro* response of the hemocyte to different stimuli as previously described [20]. Untreated hemocyte samples in ASW were run in parallel. All incubations were carried out at at 16°C utilising a cell number of about 1–2·10^6^ cells/ml (for determination of cell number, see the Flow Cytometry section). All experiments were performed at least 4 times in triplicate.

### Electron Microscopy

Hemocyte monolayers were seeded on glass chamber slides (Lab-Tek, Nunc, 177380) and treated with suspensions of n-TiO_2_ or n-ZnO in ASW (10 µg/ml) for different times (30 and 60 minutes) at 16°C. After incubation, cells were washed out in 0.1 M cacodylate buffer in ASW. Hemocytes were then fixed in 0.1 M cacodylate buffer in ASW containing 2.5% glutaraldehyde, for 30 minutes at room temperature. The cells were postfixed in osmium tetroxide for 10 minutes and 1% uranyl acetate for 1 hour. Subsequently, samples were dehydrated through a graded ethanol series and embedded in resin (Poly-Bed; Polysciences, Inc., Warrington, PA) overnight at 42°C and 2 days at 60°C. Ultrathin sections (50 nm) were cut parallel to the substrate and observed with G2 Tecnai bio-twin electron microscope (Philips, Eindhoven, The Netherlands) without additional staining. Digital images were taken with Megaview 3 CCD camera and iTEM software and processed with Adobe Photoshop CS2.

Aliquots (5–10 µl) of NP suspensions (n-TiO2 and n-ZnO 10 µg/ml ASW) were deposited on formvar and carbon-coated copper grids and allowed to settle for approximately 10 minutes. Grids were blotted dry to remove the excess and then covered with a small drop of stain (2% uranyl acetate) for 5 minutes. Grids were then blotted dry and immediately viewed at the electron microscope.

### Lysosomal Membrane Stability and Lysosomal Enzyme Release

Lysosomal membrane stability (LMS) in control hemocytes and hemocytes pre-incubated for 30 min with different concentrations of n-oxides (1,5 and 10 µg/ml) was evaluated by the Neutral Red Retention time assay as previously described [19] according to [41]. Lysosomal enzyme release was evaluated by measuring lysozyme activity in the extracellular medium as previously described [19] according to [42]. Lysozyme activity in aliquots of serum of control hemocytes and hemocytes incubated with NPs for 30 min was determined spectrophotometrically at 450 nm utilising *Micrococcus lysodeikticus.*

### Phagocytosis Assay

Phagocytosis of neutral red-stained zymosan by hemocyte monolayers was used to assess the phagocytic ability of hemocytes [43]. Neutral red-stained zymosan in 0.05 M Tris–HCl buffer (TBS), pH 7.8, containing 2% NaCl was added to each monolayer at a concentration of about 1∶30 hemocytes:zymosan in the presence or absence of different n-oxides (1,5 and 10 µg/ml), and allowed to incubate for 60 min. Monolayers were then washed three times with TBS, fixed with Baker’s formol calcium (4%, v/v, formaldehyde, 2% NaCl, 1% calcium acetate) for 30 min and mounted in Kaiser’s medium for microscopical examination with a Vanox optical microscope. For each slide, the percentage of phagocytic hemocytes was calculated from a minimum of 200 cells.

### Extracellular Oxyradical Production and Nitrite Accumulation

Extracellular generation of superoxide by mussel hemocytes was measured by the reduction of cytochrome c [43]. Hemolymph was extracted into an equal volume of TBS (0.05 M Tris–HCl buffer, pH 7.6, containing 2% NaCl). Aliquots (500 µl) of hemocyte suspension in triplicate were incubated with 500 µl of cytochrome c solution (75 µM ferricytochrome c in TBS), in the presence and absence of NPs (1, 5, 10 µg/ml) for 30 min. In a parallel set of samples, superoxide dismutase-SOD (300 Units/ml) was included to allow specific evaluation of superoxide (O_2_^−^) generation. Cytochrome c in TBS was used as a blank. Samples were read at 550 nm at different times (0 and 30 min) and the results expressed as changes in OD per mg protein.

Nitric oxide (NO) production by mussel hemocytes was evaluated as described previously [19] by the Griess reaction, which quantifies the nitrite (NO_2_^−^) content of supernatants. Aliquots of hemocyte suspensions (1.5 ml) were incubated at 16°C with NP suspensions for 0–4 h. Every 60 min, samples were immediately frozen and stored at −80°C until use. Before analysis, samples were thawed and centrifuged (12,000 *g* for 30 min at 4°C), and the supernatants were analyzed for NO_2_^−^ content. Aliquots (200 µl) in triplicate were incubated for 10 min in the dark with 200 µl of 1% (wt/v) sulphanilamide in 5% H_3_PO_4_ and 200 µl of 0.1% (wt/v) N-(1-naphthy)-ethylenediamine dihydrochloride. Samples were read at 540 nm, and the molar concentration of NO_2_^−^ in the sample was calculated from standard curves generated using known concentrations of sodium nitrite.

### Flow Cytometry

Aliquots (50 µl) from the fresh hemocyte suspensions (obtained from 8–10 individuals) were added to 250 µl of PBS-NaCl. Samples were analyzed by flow cytometry (FACScalibur, BD Becton Dickinson, San Jose, CA, USA). Data acquisition and analysis were performed with BD CellQuest software using the parameters of relative size (FSC) and granularity (SSC). Counting beads (Dako Cytocount™ ) were added in a volume of 50 µl to each tube, to allow for total hemocyte count (THC). Five gates were set up to identify the three cell sub-populations, as well as spermatozoa, cell debris, and aggregates, that were not considered for further analysis.

Aliquots of hemolymph (each containing about 1–2·10^6^ cells/ml) were incubated with different n-oxides (10 µg/ml) for 45 min at 16°C and analyzed on a FACSCalibur flow cytometer (Becton Dickinson, San Diego, CA, USA). Samples were then stained with different fluorescent probes for FC analysis. All incubations were carried out at 16°C. Hemocyte viability : aliquots of 150 µl hemolymph were incubated with propidium iodide (PI, final concentration 20 µg/ml) for 10 min and fluorescence was measured at 550–600 nm. No significant changes in hemocyte viability were observed in the different experimental conditions (data not shown).

Determination of mass/number of mitochondria: hemocytes were incubated with the mitochondrial selective dye Mito Tracker Green FM-MT (50 nM). After incubation for 30 min cells were analysed by FC on FL1 (excitation wavelength 488 nm; emission wavelength: 516 nm) as previously described [19].

Mitochondrial membrane potential (MMP or Δψ_m_) was evaluated by the fluorescent dye TMRE (Tetramethylrhodamine, ethyl ester perchlorate), as previously described [19]. Hemocytes were incubated with 40 nM TMRE for 10 min before FC analysis using an excitation wavelength of 488 nm and an emission wavelength of 580 nm.

Mitochondrial cardiolipin (CL) oxidation was evaluated by the CL sensitive probe, 10-nonyl-acridine orange (NAO) [44]. After exposure to n-oxides, cells were collected by centrifugation, washed in PBS-NaCl buffer, resuspended in the same buffer containing 100 nM NAO and incubated for 30 min. FL1 fluorescence was analysed using an excitation wavelength of 488 nm and an emission wavelength of 519 nm. To evaluate changes in fluorescence intensity (FI) values, we considered the original input on untreated cells as control (100%). Sample acquisition and analyses were performed by a FACSCalibur flow cytometer equipped with CellQuest™ software.

### Statistical Analysis

Data are the mean ± SD of at least 4 independent experiments in triplicate. Statistical analysis was performed by using ANOVA followed by Tukey’s post hoc test with significance at P≤0.05.

## Supporting Information

Figure S1 TEM images of primary particles A) n-TiO_2_; B) n-SiO_2_; C) n-ZnO; D) n-CeO_2_.
(TIF)
